# Diffusion Tensor Imaging to Analyze White Matter Tract Abnormalities in Major Psychiatric Disorders

**DOI:** 10.1055/s-0045-1814098

**Published:** 2025-12-29

**Authors:** Gunjan Jindal, Poonam Bharti, Amit Shrivastava, Shiva Kumar, Preeti Garg

**Affiliations:** 1 Department of Radiology, Maharishi Markandeshwar Institute of Medical Sciences and Research, Mullana, Haryana, India; 2 Department of Radiology and Clinical Neurosciences, University of Calgary, Alberta, Canada; 3 Department of Psychiatry, Maharishi Markandeshwar Institute of Medical Sciences and Research, Mullana, Haryana, India; 4 Department of Oral Medicine and Radiology, Maharishi Markandeshwar College of Dental Sciences, Mullana, Haryana, India

**Keywords:** diffusion tensor imaging, psychiatric illnesses, fractional anisotropy, ADC

## Abstract

**Background:**

Diffusion tensor imaging (DTI) has emerged as a valuable tool in studying depression-related brain changes. DTI is a type of magnetic resonance (MR) imaging that maps the diffusion of water molecules to visualize white matter tracts in the brain. By measuring the diffusion of water molecules in brain tissue, DTI provides information about the structural organization of white matter fibers and the integrity of neural pathways.

**Methods:**

A total of 30 cases with the first episode of severe depression were included in the study. Age- and gender-matched 30 healthy controls were also included. A structured clinical interview was conducted using the Diagnostic and Statistical Manual of Mental Disorders, fourth edition (DSM-IV) criteria for the diagnosis of major depression and the Hamilton Rating Scale for Depression was calculated. A 1.5 Tesla (T) MR System was used to determine the fractional anisotropy (FA) and apparent diffusion coefficient (ADC) values of 10 main white matter fiber tracts. Mann–Whitney U test was applied and *p* -values of all the 10 white matter fiber tracts were calculated.

**Results:**

DTI analysis found that FA values were significantly lower ( *p* < 0.05) in patients compared with healthy controls in the fornix ( *p* = 0), cingulum hippocampus ( *p* = 0.011), inferior fronto-occipital fasciculus ( *p* = 0.013), and superior longitudinal fasciculus ( *p* = 0.026). ADC values were significantly higher in the fornix ( *p* = 0.040) and reduced in anterior thalamic radiation ( *p* = 0.011).

**Conclusion:**

We concluded that various microstructural changes occur in these white matter tracts, mainly in the fronto-limbic system and play a key role in the pathophysiology of depression. This information can help in developing methods of preventing such alterations, which can reduce the morbidity of depressive patients.

## Introduction

The history of diffusion imaging dates back to the early 1990s when it was first utilized in neuroradiology for studying ischemic strokes. The first diffusion-weighted magnetic resonance (MR) sequences were described by Stejskal and Tanner. 1 With the advent of technology, the application of diffusion has expanded to other areas, such as diagnosing pyogenic infection, masses, trauma, and edema due to various causes. Today, this technique has taken a step forward with the introduction of diffusion tensor imaging (DTI) and fiber tractography, through which we can peek at the white matter connections of the brain in an entirely noninvasive manner.

These modalities have improved our understanding of ample neurologic and psychiatric illnesses, and presently, they are being utilized for mapping the white matter before any neurosurgical resections. 2 The contrast that is produced in diffusion-weighted imaging is based on the differences in the amount of diffusion of water molecules. The basic mechanism behind diffusion is the Brownian motion, which essentially means the random movement of the water molecules.

This phenomenon is dependent upon various factors, including the type of molecule, temperature, and micro-environment under study. For instance, the diffusion in cerebrospinal fluid is more as compared with that within inter- and intracellular compartments. We can choose the necessary diffusion-sensitive MR sequences to measure the differences in the magnitude of diffusion rates and eventually create the image contrast. The spatial distribution of the diffusion rate within the brain is represented on quantitative maps that are generated. Post-processing also displays an apparent diffusion coefficient (ADC) map, which is a pixel-by-pixel map. The intensity of the ADC map can be used for the quantitative estimation of the regional ADC. 3 DTI has emerged as a valuable tool in studying depression-related brain changes. The white matter fiber tract organization in the brain is estimated by DTI using anisotropic diffusion. The water molecules within the brain's white matter tend to diffuse more freely along the direction of axonal fascicles than across them. Such directional dependence of diffusivity is termed anisotropy. Data collected by DTI can be used to prepare fiber tractography, which is a 3D reconstruction technique and is used to access neural tracts. Through DTI, fractional anisotropy (FA) is calculated, which is a score ranging from 0 to 1. FA scores are calculated by calculating the movement of molecules parallel to axons and perpendicular to axons. Reduced movement results in decreased FA values, which tell us the efficiency with which neurons are signaling with each other.

DTI measures the diffusion of water molecules in brain tissue, thereby providing information on the structural orientation of white matter tracts creating a neural pathway and the integrity of white matter pathways. A study has found that some DTI metrics (e.g., FA, mean diffusivity) are associated with the severity of depressive symptoms. For instance, reduced FA, the most commonly used index of white matter integrity, in the cingulum bundle has been related to higher depressive symptomatology. 4

Depression is a leading cause of global morbidity, making it crucial that we take proactive steps now to prevent it. It not only comes with repercussions in terms of emotional and cognitive symptoms, but also structural and functional changes in the brain. In depression, structural brain changes often include reduced white matter integrity, particularly in fronto-limbic pathways. DTI demonstrates disruption of such white matter tracts, the uncinate fasciculus, and cingulum bundle. These changes affect communication between emotion-regulating regions like the prefrontal cortex and amygdala. Functionally, this can lead to faulty emotional processing and regulation. The negative impact of the depressive disease on brain structures, especially the significant impairment in white matter integrity, has been well established.

Findings from research studies have consistently shown abnormal white matter microstructural properties in depression, which reflect disturbances in the communication network of the brain nodes.

## Methods

Patients with the first episode of severe depression were included in the study. Age- and gender-matched healthy controls were taken. A total of 30 patients aged 19 to 60 years who were diagnosed with a first episode of severe depression according to Diagnostic and Statistical Manual of Mental Disorders, fourth edition (DSM-IV) criteria were included in the study. All participants included were right-handed and provided written informed consent after receiving a detailed explanation of the study. A Hindi and English patient information sheet was provided to all of the study subjects. The Maharishi Markandeshwar Institute of Medical Sciences and Research Institutional Ethical Committee approved this study. Data were collected regarding the type of family, education, marital status, socio-economic status, locality, total monthly income, total members in the family, smoking, heavy alcohol intake, and drug abuse. Revised B. G. Prasad Socioeconomic Status classification, January 2022, was used. 5 Body mass index of each participant was calculated. A detailed psychiatric evaluation was done by an experienced psychiatrist with more than 15 years of experience. Major depressive disorder (MDD) was diagnosed by taking a detailed history. A structured clinical interview was conducted using DSM-IV criteria for the diagnosis of major depression. The 17-item Hamilton Rating Scale for Depression was calculated for each patient to judge the severity of the disease. According to the 17-item Hamilton Rating Scale for Depression, severe depression was diagnosed if the patient had a score of 23 or higher. This rating score was also calculated post-8-week treatment for each patient. 6

In this study, 1.5 Tesla (T) MR System Multiva-Philips magnet (Amsterdam, the Netherlands) was used to determine FA and ADC values of 10 main white matter fiber tracts. Imaging with axial T1WSE, sagittal T2WSE, and 3D FLAIR sequences was done by means of diffusion-weighted neurography with *b* values of 0 and 800 s/mm ^2^ . Acquisition was done using an 8-channel head coil. Spin-echo DTI echo planar imaging sequence was used to acquire DTI images. Appropriate ethical clearance from the ethical committee of our institute was taken. On MR imaging (MRI), all patients who were detected with some pathology were excluded. Patients below the age of 18 years were excluded. Other exclusion criteria were a previous history of depression or any other mental disorder, Mini-Mental scale score less than 24, and presence of any metal devices which are not compatible with MRI.

## Statistical Analysis

Patient characteristics were reported using descriptive analyses. Chi-square tests were used to compare categorical demographic data between cases and controls (such as gender, level of education, or employment status), allowing assessment of whether the distributions significantly differed. The normality of the data was tested using the Shapiro–Wilk test. Associations between mean FA and mean ADC values in the different regions of interest between cases and controls were analyzed using the Mann–Whitney U test. Since some of the variables failed to satisfy the normality assumption, nonparametric Mann–Whitney U tests were employed to establish differences in mean FA and ADC values between groups. These tests resulted in the detection of whether or not significant differences in white matter microstructure existed among individuals with depression and healthy controls. The correlation between the FA values and Hamilton Rating Scale for Depression scores at baseline and post-8 weeks was analyzed using Spearman's rho correlation. A *p* -value <0.05 was considered statistically significant. All data analyses were performed using SPSS version 26.0 (SPSS, Inc.).

## Results

This study included 30 patients with a first episode of severe depression and 30 age- and gender-matched controls. The chi-square test was applied and no significant difference was found between the cases and controls. The demographic characteristics of patients were as follows: 40% of the patients were between 19 and 40 years of age, 40% were between 41 and 60 years of age, and 20% were above 61 years of age. Among the controls, 50% of the patients were between 19 and 40 years of age, 40% were between 41 and 60 years of age, and 10% were above 61 years of age. In addition, 40% of patients were male and 60% were female, whereas 46.7% of controls were male and 53.3% were female ( Table 1 ).

**Table 1:** Demographic characteristics of cases

Variable		*N* (%)
1. Age	19–40 y	12 (40.0)
	41–60 y	12 (40.0)
	>60 y	6 (2.00)
2. Gender	Male	12 (40.0)
	Female	18 (60.0)
3. Type of family	Nuclear	14 (46.7)
	Joint	16 (53.3)
4. Locality	Rural	19 (63.3)
	Urban	11 (36.7)
5. Marital status	Married	26 (86.7)
	Unmarried	4 (13.3)
6. Socio-economic status	Upper	4 (13.3)
	Upper middle	7 (23.3)
	Middle	11 (36.7)
	Lower middle	8 (26.7)
7. Smoking	Smoker	7 (23.3)
	Nonsmoker	23 (76.7)
8. Heavy alcohol use	Alcoholic	2 (6.7)
	Nonalcoholic	28 (93.3)

Overall, 46.7% patients lived in nuclear families and the rest lived in joint families. Additionally, 63.3% lived in rural surroundings and 36.7% lived in urban surroundings. Regarding socioeconomic status, 13.3% belonged to upper class; 23.3% belonged to the upper middle class; 36.7% belonged to the middle class, and 26.7% belonged to the lower middle class. Among the patients, 7 (23.3%) were smokers and 2 (6.7%) were heavy alcohol abusers ( Tables 1 and 2 ).

**Table 2:** Comparison of cases and controls in relation to demographic characteristics

Characteristics		Cases	Controls	*p* -Value a
Age	19–40 y	12 (40.0)	15 (50.0)	0.513
	41–60 y	12 (40.0)	12 (40.0)	
	>60 y	6 (2.00)	3 (10.0)	
Gender	Male	12 (40.0)	14 (46.7)	0.602
	Female	18 (60.0)	16 953.3)	

a Chi-square test.

Eight patients had suicidal thoughts and 22 patients had suicidal behavior. The baseline Hamilton Rating Scale for Depression was mild in 7 patients at the time of presentation, moderate in 16, and severe in 7 patients at the time of presentation. Post-8 weeks of treatment, the Hamilton Rating Scale for Depression improved to mild in 10 patients, moderate in 8, and severe in 2 patients ( Table 3 ).

**Table 3:** Distribution of risk factors for depression among cases

Variable		*N* (%)
Disease duration	<4 weeks	23 (76.7)
	4–8 weeks	1 (3.3)
	8–12 weeks	3 (10.0)
	>12 weeks	3 (10.0)
Presence of suicidal thoughts	Yes	8 (26.7)
	No	22 (73.3)
Presence of suicidal behaviors	Yes	1 (3.3)
	No	29 (96.7)
Family history of depression	Yes	26 (86.7)
	No	4 (13.3)
Baseline HRSD	Mild	7 (23.3)
	Moderate	16 (53.3)
	Severe	7 (23.3)
Post 8 weeks treatment HRSD	Mild	10 (33.3)
	Moderate	18 (60.0)
	Severe	2 (6.7)

Abbreviation: HRSD, Hamilton Rating Scale for Depression.

Mann–Whitney U test was applied and *p* -values of all 11 white matter fiber tracts were calculated. DTI analysis using region of interest (ROI) found out that FA values were significantly lower ( *p* < 0.05) in patients as compared with healthy controls in the following regions: fornix ( *p* = 0), cingulum hippocampus ( *p* = 0.011), inferior fronto-occipital fasciculus ( *p* = 0.013), and superior longitudinal fasciculus (SLF; *p* = 0.026; Table 4 ).

**Table 4:** Comparison of the mean FA values in the different regions of interest between cases and controls

Region of interest	FA cases (mean ± SD)	FA controls (mean ± SD)	*p-* Value a
Fornix	0.378 ± 0.046	0.455 ± .048	0.00 b
Anterior thalamic radiation	0.467 ± 0.031	0.465 ± 0.042	0.69
Arcuate fasciculus	0.496 ± 0.038	0.488 ± 0.041	0.762
Cingulum cingulate	0.419 ± 0.042	0.509 ± 0.055	0.201
Cingulum hippocampus	0.419 ± 0.054	0.456 ± 0.040	0.011 b
Corticospinal tract	0.561 ± 0.089	0.533 ± 0.026	0.076
Inferior fronto-occipital	0.490 ± 0.055	0.550 ± 0.101	0.013 b
Inferior longitudinal fasciculus	0.473 ± 0.032	0.507 ± 0.108	0.026 b
Forceps major	0.530 ± 0.045	0.512 ± 0.039	0.126
Forceps minor	0.529 ± 0.043	0.498 ± 0.061	0.089
Uncinate fasciculus	0.454 ± 0.039	0.473 ± 0.039	0.068

Abbreviations: FA, fractional anisotropy; SD, standard deviation.

a Mann–Whitney U test.

b *p* < 0.05.

Our analyses also revealed significantly reduced ADC values in the anterior thalamic radiation (ATR) and corticospinal tracts in patients with severe depression, as well as increased ADC values in the fornix with *p* -value of <0.05 ( Table 5 ).

**Table 5:** Comparison of the mean ADC values in the different regions of interest between cases and controls

Region of interest	ADC cases (mean ± SD)	ADC controls (mean ± SD)	*p-* Value a
Fornix	1.28 ± 0.291	1.126 ± 0.255	0.040 b
Anterior thalamic radiation	0.855 ± 0.052	0.915 ± 0.109	0.011 b
Arcuate fasciculus	0.801 ± 0.046	0.814 ± 0.040	0.145
Cingulum cingulate	0.827 ± 0.051	0.815 ± 0.048	0.228
Cingulum hippocampus	0.956 ± 0.147	0.920 ± 0.088	0.600
Corticospinal tract	0.808 ± 0.111	0.863 ± 0.054	0.022 b
Inferior fronto-occipital	0.826 ± 0.075	0.826 ± 0.039	0.569
Inferior longitudinal fasciculus	0.864 ± 0.101	0.853 ± 0.031	0.988
Forceps major	0.909 ± 0.081	0.883 ± 0.069	0.178
Forceps minor	0.849 ± 0.056	0.835 ± 0.040	0.403
Uncinate fasciculus	0.861 ± 0.063	0.845 ± 0.051	0.433

Abbreviations: ADC, apparent diffusion coefficient; SD, standard deviation.

a Mann–Whitney U test.

b *p* < 0.05.

Our analyses found a significant reduction in FA values of the hippocampus and parahippocampal region in cases as compared with controls. However, no correlation was found between FA and ADC of cingulum gyrus and depression. A similar study conducted by Srivastava et al in 2016 showed lower FA values in the hippocampus. 7 This study involved 15 MDD patients and 15 healthy controls without intergroup differences in age, gender, or education. ROI analysis demonstrated significantly lower FA values in MDD patients in some brain regions, including the left SLF, right lentiform, right hippocampus, and left inferior parietal cortex. Statistically significant differences in FA values of cingulum cortex were reported in a study conducted by Korgaonkar et al and they also reported that these fiber tracts can be used as predictors of treatment. 8 However, few studies have found no significant correlation between the FA values in the bilateral parahippocampal gyrus region and depression. 9 The limbic system of the brain is responsible for emotions, behavior, memory, and motivation. Various studies conducted previously have found that anterior cingulate–limbic white matter is altered in patients with severe depression, as predicted in our study. 7 8 Therefore, this white matter fiber tract can be used to predict treatment outcome and severity of depression in patients.

Our analyses also demonstrated reduced FA values and increased ADC values in the fornix.

The fornix consists of major white matter fibers that pass through the hippocampus, too, therefore, validating this finding. Similar results were determined by another study conducted in 2016. 10

The ATR is a significant white matter tract that connects the dorsolateral prefrontal cortex—a region critical for executive function, decision-making, and working memory—with the anterior and dorsomedial thalamic nuclei. These nuclei play essential roles in attention, alertness, and motivation. These fibers traverse the anterior limb of the internal capsule, a major white matter tract that serves as a conduit for subcortical–cortical communication. In addition to planning and executive operations, the ATR is also implicated in emotional control and goal-directed behavior due to its role in the thalamo-prefrontal circuitry. Disruption of ATR integrity has been associated with several psychiatric and neurodegenerative conditions, like MDD, schizophrenia, and Alzheimer's disease. Previous studies have documented a correlation between reduced FA values in ATR and depression. 11 12 However, we did not find any conclusive evidence to suggest a correlation between FA values of ATR and depression. However, reduced ADC values were found in ATR in our study. A study conducted by Niida et al demonstrated significantly higher ADC values in ATR in Alzheimer's disease patients as compared with MDD patients. 13

The inferior fronto-occipital fasciculus (IFOF) is a long association fiber bundle that begins in the parietal and occipital lobes, traverses anteriorly lateral to the insula via the extreme external capsule, and terminates in the inferior frontal lobe along the opercular gyri. 14 Our study also demonstrated that FA values are lower in IFOF in patients with major depression as compared with healthy controls. Similar results were concluded by Sugimoto et al, who demonstrated that the FA values of the bilateral IFOF and corpus callosum genu were significantly lower in patients with severe depression. 15 Degeneration of this white matter fiber tract has also been demonstrated in patients with several other neuropsychiatric behavioral disorders, such as antisocial personality disorder and obsessive-compulsive disorder. No significant alteration in ADC values was found in IFOF in our study.

Many studies have been conducted that 8 16 revealed significantly lower FA values in the SLF 17 in patients with severe depression, which was confirmed in our study as well. SLF is the largest association fiber of the brain and it connects the perisylvian areas in the ipsilateral frontal, parietal, and temporal lobes.

However, previous studies have also demonstrated lowered FA values in the parieto-occipital region, which was not validated by our study. Wu et al demonstrated significantly lowered FA values in the left parieto-occipital region in single-episode treatment-naïve MDD patients. 18 Srivastava et al also demonstrated lowered FA values in parieto-occipital white matter fiber tracts, which was not validated in our study. 7

The limitations of our study were that our study population may not represent the entire population, as all patients were from the north Indian region. The cases and controls were age- and gender-matched only and not matched on any other parameter like socio-economic status.

## Conclusion

Our study has demonstrated a correlation between lower FA values and depression in white matter fiber tracts of the cingulum hippocampus, fornix, IFOF, and SLF. This shows that various microstructural changes occur in various white matter tracts, mainly in the fronto-limbic system, and play a key role in the pathophysiology of depression. Our study also demonstrated a correlation between lowered FA values and SLF, which is the largest association fiber. These findings are significant as they will help researchers to further develop methods and tools to diagnose changes in white matter tracts in depressive patients, which can be of diagnostic and therapeutic value. Research can be done on the prevention of such alterations from occurring, which can reduce the morbidity of depressive patients.
